# Comparing Intranasal Midazolam, Oral Melatonin, and Distraction Cards for Pain and Stress Management in Pediatric Intravenous Line Insertion: A Randomized Controlled Trial

**DOI:** 10.1155/prm/9887917

**Published:** 2026-02-11

**Authors:** Sima Akhavan, Sedighe Shah Hosseini, Nilufar Amini, Minoo Saeidi

**Affiliations:** ^1^ Pediatric Pulmonologist, Imam Hossein Children’s Hospital, Isfahan University of Medical Sciences, Isfahan, Iran, mui.ac.ir; ^2^ Assistant Professor of Pediatric Anesthesiology, Imam Hossein Children’s Hospital, Department of Anesthesiology, Isfahan University of Medical Sciences, Isfahan, Iran, mui.ac.ir; ^3^ Anesthesiology and Critical Care Research Center, Isfahan University of Medical Sciences, Isfahan, Iran, mui.ac.ir; ^4^ Assistant Professor of Pediatrics, Imam Hossein Children’s Hospital, Department of Pediatrics, Isfahan University of Medical Sciences, Isfahan, Iran, mui.ac.ir; ^5^ Interprofessional Group of Palliative Care, Isfahan University of Medical Sciences, Isfahan, Iran, mui.ac.ir

**Keywords:** child, distraction, interventions insertion, melatonin, midazolam, pain, stress

## Abstract

**Background:**

Intravenous (IV) line insertion causes pain and stress in children. However, we know about the permanent effects of painful experiences on a child’s life, but this main clinical complaint has been underestimated and left untreated. This study was conducted to compare the effect of the nonpharmacological method of “distraction” and two medicinal methods, “nasal midazolam” and “oral melatonin,” on reducing pain and stress caused by IV line insertion in children.

**Methods:**

Patients were randomly assigned to one of the four groups: control, distraction, oral melatonin, and nasal midazolam. Pain and stress scores were measured and compared using a standard questionnaire during and after IV insertion.

**Results:**

In this study, the partial *η* ^2^ = 0.12 for stress and 0.085 for pain. Parental presence showed a stronger effect (*p* value < 0.05), with a partial *η* ^2^ = 0.27 in reducing stress and 0.30 in reducing pain. The underlying disease of the child had no significant relationship with pain and anxiety (*p* value > 0.05).

**Conclusion:**

In this study, we found that the levels of stress and pain were statistically significant between the 4 study groups: the children who had not managed to reduce pain and anxiety before IV insertion (control group), the oral melatonin group, the nasal midazolam group, and the distraction group (*p* value = 0.016 and *p* value = 0.002, respectively). Oral melatonin is more effective than nasal midazolam in reducing pain and stress caused by venipuncture in children.

**Trial Registration:**

Iranian Registry of Clinical Trials (IRCT): 20220128053852N2

## 1. Introduction

Procedural pain and related anxiety can have profound immediate and long‐term effects on children, ranging from behavioral and psychological distress to increased healthcare avoidance in later life [1]. Contemporary systematic reviews, meta‐analyses, and clinical toolkits all emphasize that effective procedural pain management improves not only patient comfort but also family satisfaction and cooperation and reduces emotional trauma [2, 3]. However, in resource‐limited environments like ours, the application of pharmacological approaches is fraught with safety concerns due to personnel shortages [4]. As a result, nonpharmacological techniques—such as distraction, play‐based interventions, and cognitive‐behavioral strategies—may offer feasible, minimal‐risk alternatives for procedural pain control [5].

Recent high‐quality studies have highlighted the comparative efficacy of various interventions. Pharmacological agents, including intranasal midazolam and oral melatonin, are supported by recent clinical trials and meta‐analyses as effective for reducing procedural pain and anxiety in children [4]. Likewise, distraction cards and other nonpharmacological modalities are increasingly recognized for their practicality and positive effect on pain and stress, especially when pharmacological interventions are not feasible due to resource or safety constraints [5, 6]. Despite the strong evidence base, uptake of such interventions in our hospital—and in similar centers with staffing limitations—remains unsatisfactory.

Despite increasing global awareness and evolving guidelines on pediatric procedural pain management, the reality in many hospitals—including Imam Hussein Children’s Hospital—remains that pain control for children is often not prioritized in routine clinical practice. Organizational focus at our center has historically overlooked both pharmacological and nonpharmacological strategies for alleviating procedural pain in children. A critical limitation is the lack of adequately trained anesthesiologists and anesthesia technicians, which creates significant challenges for the safe use of sedative and analgesic drugs, leaving many pediatric patients vulnerable to untreated pain and distress during routine interventions such as intravenous cannulation [7].

We hypothesized that oral melatonin and intranasal midazolam would reduce pain and stress scores during pediatric IV insertion more effectively than distraction cards or no intervention (primary hypothesis). Additionally, we hypothesized superior first‐attempt success rates and parental satisfaction in pharmacological groups compared to distraction and control (secondary hypotheses).

### 1.1. Study Design and Participants

This study was a randomized controlled trial. This study obtained scientific code and ethics approval from the Ethics Committee of the Faculty of Medicine of Isfahan University of Medical Sciences (IR.MUI.MED.REC.1400.696). In this study, we enrolled children aged 3–10 years who underwent pediatric intravenous line insertion in the emergency department of Imam Hossein Children’s Hospital in Isfahan between April 2023 and December 2023. The inclusion criteria include any child, who vomits and does not tolerate oral rehydration therapy, aged 3–10 years with mild to moderate dehydration related to gastroenteritis. Also, the exclusion criteria include any visual or auditory defects with difficulty in communication with the child; parental refusal to continue the process; acetaminophen and other analgesic use in 6 previous hours; ASA classification of 3 or higher; need for urgent intravenous access due to severe dehydration or any other causes; personal or family history of previous adverse reaction to sedative agents; high‐risk children for aspiration, such as untreated gastroesophageal reflux disease or other GI motility disorders; and any uncontrolled severe underlying diseases, such as cardiac or neurologic disorders. The sample size was calculated using Epi Info software, with a 95% confidence interval and 80% power. This resulted in a sample size of 30 patients per group. This study adheres to CONSORT guidelines, and a completed CONSORT checklist is available as a supplementary file.

### 1.2. Randomization and Intervention

Upon arrival at the emergency department, a trained pediatric assistant screened potential participants for eligibility based on predefined inclusion and exclusion criteria. Following this screen, the assistant engaged in a conversation with the parents to provide them with information about the research study, including its objectives, and to address any questions or concerns they may have had. Verbal informed consent was then obtained from the parents.

Following informed consent, demographic data, vital signs, medical history, and initial physical examination findings were collected and documented for enrolled participants.

Participants were randomly assigned to one of four intervention groups (control, distraction, melatonin, and midazolam) using a computer‐based service at https://www.sealedenvelope.com, with a block randomization method. This method ensures balanced group sizes throughout enrollment. The sequence generation involved creating random blocks of assignments that were predetermined before participant enrollment began. This approach minimizes selection bias and maintains comparable group sizes for valid statistical comparisons.

Allocation concealment was maintained by having a trained pediatric assistant responsible for the group assignments. The randomization sequence was prepared in advance but concealed from the research team enrolling participants and conducting assessments. The pediatric assistant did not disclose the upcoming group assignments to the enrollment personnel until after participants had been deemed eligible and had consented, which prevented foreknowledge of the next assignment and protected against selection bias.

Due to the nature of the interventions (distraction cards, oral melatonin, intranasal midazolam, and control with no intervention), blinding of participants and care providers was not fully feasible. Parents and children were aware of their assigned intervention group because of obvious differences in the procedures. However, to reduce potential assessment bias, outcome assessors who recorded pain and stress scores were blinded to the participants’ group assignment where possible. Where blinding was not possible, this limitation and its potential impact on study findings were acknowledged.

Pain and stress levels were assessed using validated, age‐appropriate scales to ensure reliable and standardized measurement. For pain assessment, we employed the Wong‐Baker FACES Pain Rating Scale and FLACC scale, which are widely used and validated for pediatric populations [8]. Stress was measured using the Children’s Emotional Manifestation Scale (CEMS), known for its reliability in children undergoing medical procedures [9]. Both the pain and stress scoring scales were administered to each participant throughout the intravenous cannulation procedure (during) and upon completion (after) at 5 and 15 min postprocedure. Additionally, for participants in the midazolam and melatonin intervention groups, vital signs were monitored at defined intervals until full recovery.

To minimize potential bias in outcome assessment, the individuals responsible for measuring pain and stress scores were blinded to the group allocation of the participants. The assessor had no involvement in the randomization process or intervention administration. Participants and their caregivers were instructed not to disclose the assigned intervention during the evaluation. Additionally, allocation concealment was maintained by having a separate pediatric assistant perform group assignments without disclosing them to the research staff conducting outcome measurements. These measures helped reduce detection and selection bias, thereby increasing the validity of our findings.

**Figure 1:**
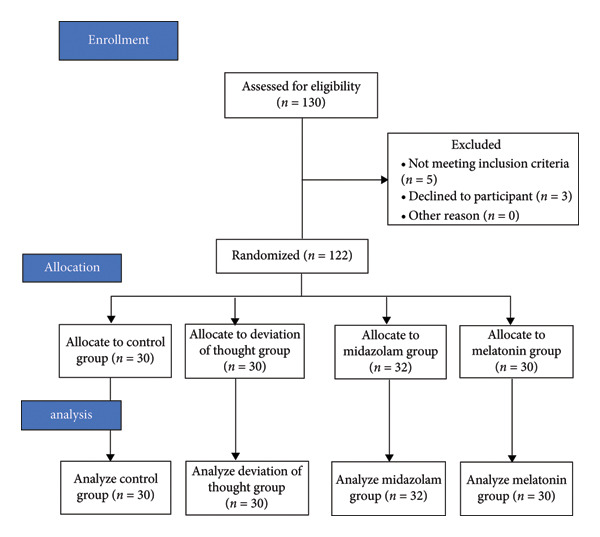
The process of the study according to the CONSORT flow diagram.

Finally, 122 patients were randomly allocated to one of the four intervention groups: a control group, a distraction group, a nasal midazolam group, and an oral melatonin group, each comprising 30 children. The CONSORT flow diagram (Figure 1) illustrates the allocation. The 4 intervention groups included the following.

#### 1.2.1. Group 1: Distracting the Child With Distraction Cards

**Figure 2:**
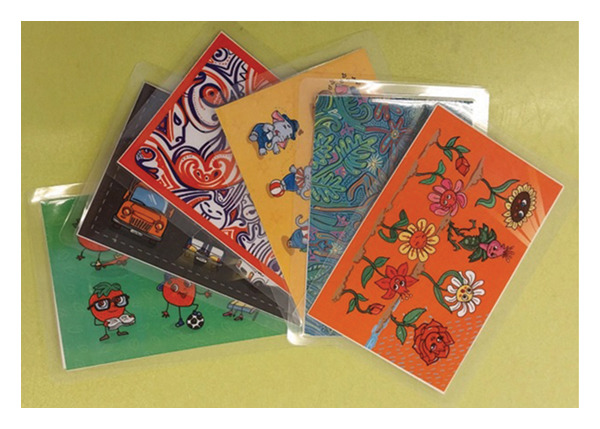
Author designed distraction cards.

The distraction method was performed by introducing standard picture cards (Figure 2) to the child by a trained nurse 3 min before and during intravenous cannulation.

#### 1.2.2. Group 2: Administration of Intranasal Midazolam

Midazolam was administered at a dose of 0.2 mg/kg and with a maximum total volume of 0.5 cc inside one of the nasal cavities. Then, it blocked that cavity for 30 s, 15 min before the intravenous line insertion.

#### 1.2.3. Group 3: Administration of Oral Melatonin

It was done by administering 0.5 mg/kg (maximum dose of 5 mg) of melatonin orally, 30 min before inserting the intravenous line.

#### 1.2.4. Group 4: Control Group

In the control group, no member of the research team had any interaction with the child. Parental presence involved no verbal coaching, play, or distraction during the procedure to better isolate the effect of the intervention. In all 3 intervention methods, at least one parent has accompanied the child.

### 1.3. Statistical Analysis

Data were entered into SPSS (v. 23), checked for missing values, and summarized using descriptive statistics. Group differences in baseline characteristics were tested using Chi‐square tests for categorical variables, one‐way ANOVA for parametric continuous variables, and Kruskal–Wallis tests for nonparametric continuous variables. To evaluate changes in pain and stress scores over time, a GLM repeated measures ANOVA was applied to examine the effects of time, group, and their interaction, with partial eta‐squared (*η* ^2^p) reported as the effect size. Where significant effects were detected, Bonferroni‐adjusted post hoc tests were used for parametric data, and Dunn’s test with Bonferroni correction was applied for nonparametric data. The results are presented with 95% confidence intervals, where applicable.

## 2. Results

One hundred and twenty‐two children were included in this study, including 84 (68.9%) boys and 38 (31.1%) girls. There is no significant difference in gender distribution among the 4 studied groups (*p* = 0.379) (Table 1).

**Table 1:** Demographic and clinical characteristics of participants.

Variables	Group				*p* value
	Control N = 30	Deviation of thought. *N* = 30	Midazolam N = 32	Melatonin N = 30	
	N (%)/mean ± SD				
Gender					*p* = 0.379
Male	21 (70)	17 (56.7)	23 (71.9)	23 (76.7)	
Female	9 (30)	13 (43.3)	7 (28.1)	7 (23.3)	
Age	6.21 ± 2.92	5.36 ± 2.34	5.54 ± 2.27	5.55 ± 2.36	*p* = 0.662
History of IV^∗^	21 (70)	10 (66.7)	25 (78.1)	20 (66.7)	*p* = 0.724
IV try					
First attempt	24 (80)	27 (90)	31 (96.9)	25 (83.3)	*p* = 0.185
Second attempt	6 (20)	3 (10)	1 (3.1)	5 (16.7)	
History of surgery^∗^	3 (10)	9 (30)	5 (15.6)	3 (10)	*p* = 0.119
Drug history^∗^	10 (33.3)	9 (30)	6 (18.8)	3 (10)	*p* = 0.120
Underlying disease^∗^	12 (40)	13 (43.3)	4 (12.5)	7 (23.3)	*p* = 0.026
Need to parents^∗^	15 (50)	7 (23.3)	15 (46.9)	6 (20)	*p* = 0.022

^∗^Yes.

The mean ± SD of age was 5.66 ± 2.47 years (range: 3–10 years). The Kruskal–Wallis test’s results indicated no age differences among the groups (*p* = 0.662) (Table 1).

The demographic and clinical characteristics of the participants are shown in Table 1. Based on the results of this table, there is no significant difference in the history of IV in the studied groups (0.724). Also, intravenous cannulation was successfully performed in 170 (87.7%) cases in the first attempt. In 15 (12.3%) cases, the first attempt was unsuccessful, and a second attempt was performed. In the control group, in 20% of the cases, the child’s intravenous line was not successful in the first attempt, and these percentages were 17% and 10% in the midazolam and distraction groups, respectively. In the melatonin group, 97% of cases were completed on the first attempt, and only 3% required a second try. A total of 15 children were reported to reinsert the IV line, the highest (40%) in the control group and the lowest (7%) in the melatonin group.

On the other hand, the results show that the control and deviation of thought groups exhibited a significantly higher prevalence of underlying diseases compared to the other groups (*p* = 0.026).

Furthermore, it was found that the need for parental assistance was notably greater in the deviation of thought and midazolam groups compared to the control and melatonin groups (*p* = 0.022). Other variables did not show significant differences.

In addition, the pain scores in four groups were measured during and after intravenous line insertion, and the results are presented in Table 2. The results showed that all groups experienced a significant increase in pain score after intravenous line insertion; the rate of increase differed significantly between groups (*p* < 0.0001). Specifically, the control group exhibited a significantly greater rise in pain score compared to Group 2 (*p* = 0.002). Interestingly, the pain score increases over time (during and after intravenous line insertion), whereas the other groups did not show statistically significant differences.

**Table 2:** Comparison of pain scores over time across 4 groups.

Group time	Control	Deviation of thought	Midazolam	Melatonin	*p* value^∗^
	Mean ± SD of pain scores				
During intravenous line insertion	3.03 ± 3.06	3.80 ± 3.79	4.09 ± 4.41	2.57 ± 2.82	*p* = 0.866
After intravenous line insertion	6.23 ± 3.61	5.93 ± 4.04	5.31 ± 4.33	4.7 ± 3.26	*p* = 0.360
Mean difference	3.2 ± 2.91	2.13 ± 2.28	1.21 ± 1.45	2.13 ± 2.34	*p* = 0.012
*p* value	*p* < 0.0001	*p* < 0.0001	*p* < 0.0001	*p* < 0.0001	

	**Comparisons over time between groups**				
	**Mean square**	***F***	**p** **-value**	**Effect size** ^**^**^	**CI for** **η** ^2^ **p**

4 groups	17.53	3.44	0.019	0.081	[0.002, 0.152]
Control and deviation of thought	12.96	1.95	0.168	0.033	[0.001, 0.098]
Control and midazolam	51.6	10.23	0.002	0.148	[0.032, 0.245]
Control and melatonin	20.46	3.06	0.85	0.051	[0.001, 0.132]
Deviation of thought and midazolam	12.2	3.44	0.068	0.055	[0.000, 0.136]
Deviation of thought and melatonin	8.43	2.3	0.134	0.038	[0.000, 0.115]
Midazolam and melatonin	0.445	0.085	0.771	0.001	[0.000, 0.003]

^∗^Comparison of 4 groups and comparison over time.

^Partial eta‐squared (*η* ^2^p).

Also, Table 3 reports the stress scores in four groups during and after intravenous line insertion. The results indicate that intravenous line insertion resulted in a significant increase in stress scores across all groups (*p* < 0.0001). Furthermore, the stress score in the control group increased significantly more than all treatment groups over time (during and after intravenous line insertion), while the other groups did not show significant differences from each other (Table 3).

**Table 3:** Comparison of stress scores over time across 4 groups.

Group time	Control	Deviation of thought	Midazolam	Melatonin	*p* value^∗^
	Mean ± SD of stress scores				
During intravenous line insertion	11.07 ± 6.17	12.83 ± 7.45	13.0 ± 8.0	9.73 ± 4.92	*p* = 0.878
After intravenous line insertion	17.23 ± 7.21	15.83 ± 7.67	15.0 ± 8.28	12.37 ± 5.76	*p* = 0.154
Mean difference	6.16 ± 5.67	3.0 ± 3.06	2.0 ± 2.87	2.63 ± 3.46	*p* < 0.0001
*p* value	*p* < 0.0001	*p* < 0.0001	*p* < 0.0001	*p* < 0.0001	

	**Comparisons over time between groups**				
	**Mean square**	***F***	**p** **value**	**Effect size** ^**^**^	**CI for** **η** ^2^ **p**

4 groups	100.78	6.65	< 0.0001	0.146	[0.043, 0.235]
Control and deviation of thought	127.28	6.25	0.015	0.099	[0.012, 0.185]
Control and midazolam	239.004	12.19	< 0.0001	0.171	[0.058, 0.270]
Control and melatonin	209.22	9.69	0.003	0.145	[0.042, 0.234]
Deviation of thought and midazolam	15.27	1.72	0.194	0.028	[0.008, 0.104]
Deviation of thought and melatonin	4.14	0.385	0.537	0.007	[0.002, 0.019]
Midazolam and melatonin	3.8	0.373	0.544	0.006	[0.001, 0.021]

^∗^Comparison of 4 groups and comparison over time.

^Partial eta‐squared (*η* ^2^p).

**Figure 3:**
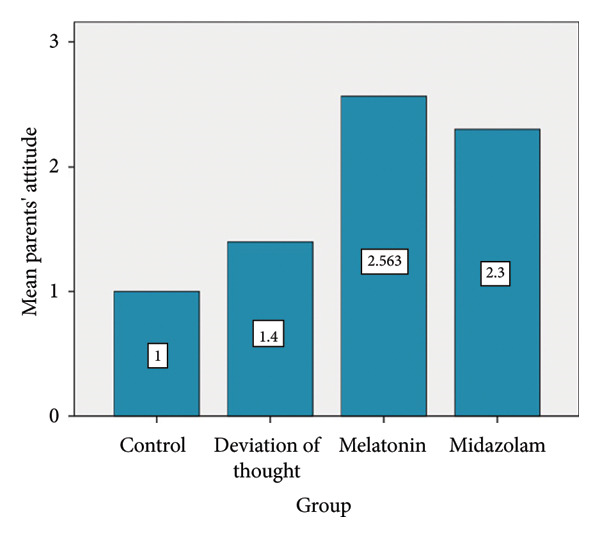
Comparing the mean score of parent’s satisfaction with treatment methods.

In this study, parents’ satisfaction with treatment methods was assessed, revealing the highest satisfaction in the melatonin group, followed by the midazolam group. Conversely, the control group exhibited the lowest satisfaction level (Figure 3). Notably, a significant difference in satisfaction was observed between the control group and both groups: melatonin (*p* < 0.0001) and midazolam (*p* = 0.001).

## 3. Discussion

Our study demonstrated that oral melatonin and distraction cards offer significant benefits for pain and anxiety management during intravenous line insertion in children, particularly compared to standard care. These interventions are especially valuable in resource‐limited pediatric emergency settings where pharmacological sedation may not always be feasible or safe due to limited staff and monitoring capacity.

Needle pain is the most common source of pain for children [10, 11]. The most common procedure in the emergency department is intravenous cannulation [12]. During acute hospitalization, time limitations make it difficult and more stressful for the child and parents to adapt to circumstances [13]. However, intravenous catheterization is in the category of a low‐pain procedure. Studies show that children usually sense their pain as moderate to severe [8, 12]. There are many straightforward guidelines for pain assessment and control in the field of pediatrics, but we are not eager to use them in our clinical practices [9, 10]. Nonpharmacological pain control modalities are inexpensive, accessible, and safe [14, 15]. Distraction is one of these methods [13, 16].

There are many studies on the effectiveness of various forms of distraction in anxiety and pain management in children, but they are not powerful studies [13]. Inan and Inal compared verbal parental interaction, video games, and cartoon movies and showed video games as a better way to control pain [17]. In another study, distraction cards performed better in comparison with a kaleidoscope [18]. Also, Ballard et al. displayed the effectiveness of distraction cards [19]. Susam et al. showed that the Buzzy system (local cryotherapy plus vibration) is more effective in conjunction with distraction cards [20].

Recent randomized controlled trials confirm our findings on melatonin [21]. Administering 0.5 mg/kg of oral melatonin 30 min before venipuncture substantially reduces procedure‐related pain and anxiety, with intervention groups experiencing lower pain and anxiety scores and a higher proportion of successful cannulation attempts compared to controls [21, 22]. Melatonin’s anxiolytic and antinociceptive properties have now been validated in several pediatric procedural and surgical contexts, consistently showing a favorable safety profile and minimal side effects [22, 23]. While some meta‐analyses report midazolam’s superiority for sedation and separation anxiety in preoperative settings, evidence also supports melatonin’s reliability and fewer adverse events, supporting its use especially where sedation depth or rapid onset is less critical [24].

Midazolam is an anxiolytic and sedative drug, and its use in children is very common. Intranasal midazolam is effective in controlling procedural anxiety control [25]. Kaiser et al. examined the effectiveness and feasibility of different intranasal volumes of midazolam [26]. In another study, intranasal midazolam was efficient and safe in children aged 2–8 years [27].

Pain management in children is often overlooked, even though clear guidelines exist. In our emergency department, we routinely assess and record children’s pain, but treatment options are rarely offered. Pain control has been viewed as a specialized task, and analgesics are underprescribed, even for hospitalized patients.

This study was conducted to address these gaps. We educated the nurses on pediatric pain assessment and highlighted the importance of addressing even mild pain. We emphasized that effective pain control is a child’s right, not just an option.

Parents responded positively to education about pain control, with 98.3% reporting satisfaction. All groups in our study experienced increased pain and stress after procedures; however, both midazolam and distraction cards were equally effective in pain control, while melatonin showed strong benefits in reducing pain and stress.

Our findings emphasize the clinical relevance of melatonin as a safe, accessible, and effective option for procedural pain and anxiety reduction in children, especially where barriers to traditional sedation exist. Expanding the use of both melatonin and structured distraction interventions should be considered part of best practice in pediatric emergency and procedural care.

Limitations of our study include a relatively small sample size and potential variability in pain perception or reporting across different cultural groups. Future research should focus on larger multicenter studies to confirm these results and explore optimal implementation pathways for nonopioid, parent‐approved interventions in procedural pediatric care.

## 4. Conclusion

This study demonstrated that distraction methods (such as distraction cards used in this study), nasal midazolam, and oral melatonin are effective in managing children’s pain and stress during IV line placement. The results showed that oral melatonin was more effective than nasal midazolam in lowering the average scores of pain and stress. One limitation of this study was the small sample size. A larger sample size could allow for more accurate comparisons. Additionally, psychological differences among Iranian families and their attitudes toward pain and painful medical procedures may have introduced bias, and we are unsure how to minimize these effects on the results.

## Conflicts of Interest

The authors declare no conflicts of interest.

## Author Contributions

Minoo Saeidi: conceptualization, project administration, funding acquisition, methodology, supervision, formal analysis, and writing–review and editing; Sima Akhavan: investigation and writing–original draft; Nilufar Amini: investigation and writing–original draft; Sedighe Shah Hosseini: methodology, investigation, and writing–review and editing.

## Funding

This research did not receive any specific grant from funding agencies.

## Data Availability

The datasets used and/or analyzed during the current study are available from the corresponding author upon reasonable request, provided that the individual’s privacy can be protected.
